# Endometriosis immune microenvironment timing shifts: from immune escape to immune exhaustion

**DOI:** 10.1038/s41420-026-02965-8

**Published:** 2026-02-26

**Authors:** Weisen Fan, Zhai Fengting, Zhao Ruihua

**Affiliations:** 1 Department of Gynecology, Guang ‘anmen Hospital, China Academy of Chinese Medical Sciences, Xicheng District, Beijing, China; 2 Department of Gynecology, Affiliated Hospital of Shandong University of Traditional Chinese Medicine, Jinan, Shandong China

**Keywords:** Urogenital reproductive disorders, Immune cell death

## Abstract

Endometriosis(EMS) symptoms, progression, and onset are all linked to the patient’s immune system dysfunction. Research on immune cells and their molecular mechanisms has long been a prime focus of EMS studies. Nonetheless, the immune environments of EMS patients and cells are constantly changing. How to effectively study and treat EMS depends on our ability to comprehend the temporal changes in immunity in EMS patients. Consequently, the immunological time sequence of endometriosis is methodically discussed and summarized in this article as four steps: 1) immune escape, in which Ectopic Endometrial Cells(EECs) evade immune surveillance and growth in the peritoneum; 2) pro-inflammatory immune response, immune killer cells are triggered, and the lesions show signs of inflammation; 3) immunological anti-inflammatory, which converts pro-inflammatory micro-environment to anti-inflammatory micro-environment and helps the body avoid excessive inflammation; 4) Immune exhaustion: Immune killer cells gradually lose their ability to attack EECs as they experience exhaustion and paralysis. In addition to providing a reference for future EMS staging treatments, this study offers a comprehensive understanding of the temporal alterations in EMS. It reveals cellular and molecular processes associated with immune exhaustion.

## Facts

- Endometriosis cells can reduce the immune recognition by immune killer cells. Therefore, it remains it is unknown whether the decline in the recognition function of immune killer cells in endometriosis occurs before or after the onset of the disease.
- Although repeated damage and repair in endometriosis can cause the immune system to shift towards an anti-inflammatory state, the relationship between the immune microenvironment and the menstrual cycle remains unknown.
- The immune microenvironment of endometriosis exhibits immune exhaustion, but there is a significant lack of research on the exhaustion of immune killer cells, which is worth further exploration.

## Introduction

A prevalent clinical condition in obstetrics and gynecology, endometriosis(EMS) can cause malignant transformation in addition to dysmenorrhea, infertility, and chronic pelvic pain. Although retrogradation of menstrual blood, body cavity metaplasia, lymphatic dispersion, and other factors are linked to the start of EMS, the pathophysiology of EMS remains unclear [1]. The ambiguity of the pathogenesis makes it challenging for us to eradicate this illness. Notably, immunological factors are linked to ectopic endometrial cells(EECs) implantation and survival, increasing lesion size and recurrence following resection [2, 3]. The inflammatory microenvironment and immune cells are dynamic in the etiology and development of EMS. It is helpful to comprehend the intricate cellular and molecular mechanisms of EMS-related immunity by knowing how the immunological microenvironment changes as EMS lesions develop. The inflammatory microenvironment and immune cells are dynamic in the etiology and development of EMS. It is helpful to comprehend the intricate cellular and molecular mechanisms of EMS-related immunity by knowing how the immunological microenvironment changes as EMS lesions develop. EECs and immune cells have a complex, ongoing communication and interaction that can both activate immunosuppressive cells and inhibit immune killer cells. We summarize the cellular and molecular mechanisms from four stages: immune escape of EECs, an early stage characterized by immune pro-inflammation in the microenvironment, a late stage marked by anti-inflammation microenvironment and immune killer cell exhaustion.

## EECs undergo immune escape

### Immune killer cell dysfunction enables EECs to escape immune surveillance

While the exact pathophysiology of EMS is currently unknown, the main clue is that immune cells do not kill EECs. Because EECs directly inhibit immune killer cells, the immune killer cells’ capacity to eliminate aberrant cells is impaired. Natural killer (NK) cells, macrophages, and cytotoxic T lymphocytes (CD8 + T) are the primary killer cells. These cells’ diminished capacity to kill contributes to the immune escape of EECs. NK cells and macrophages are the primary cells that carry out killing in the early stages [4]. NK cell cytotoxicity is reduced in EMS patients, according to multiple studies [5, 6].Compared to healthy women, EMS patients reduced NK cell cytotoxicity in both peripheral blood and abdominal cavity. Subsets of NK cells that expressed more CD16 and less CD56 were generally more cytotoxic. In general, NK cell subsets with high CD16 expression and low CD56 expression were more toxic. NK cells in peripheral blood were more toxic, and CD16brightCD56dimNK cells were the main subset. In contrast, NK cells in endometrium and peritoneal cavity were less toxic, and CD16dimCD56brightNK cells were the main subset, and their function was mainly to produce cytokines [7]. The peripheral blood of patients with EMS shows an increase in CD16dimCD56bright NK cell number, and a decrease in the CD56dimCD16bright/CD56brightCD16dim ratio [8]. Whereas NK cells in the endometrium and peritoneal cavity were less toxic and the primary subset was CD16dimCD56brightNK cells, which mostly produced cytokines, NK cells in peripheral blood were more cytotoxic and the primary subset was CD16brightCD56dimNK cells.In individuals with EMS, the CD16dimCD56bright/CD56brightCD16dim ratio dropped while the peripheral blood’s CD16dimCD56bright NK cell count rose. This suggests that the NK cells seen in EMS patients’ peripheral blood are primarily less cytotoxic subsets. Patients with EMS had lower levels of CD56 expression on NK cells in the peritoneal cavity, which could be because of EECs, which causes NK cells to change to a subgroup that is more cytotoxic [9, 10].

Nonetheless, some research has demonstrated that EMS patients’ peritoneal cavities do not exhibit changed CD56 expression of NK cells [11]. Despite changes in NK cell subsets, toxicity is primarily determined by the balance between NK cell activating and inhibitory receptors. EMS patients exhibit increased expression of inhibitory receptors on NK cells, including immunoreceptor tyrosine—based inhibition motif—killer immunoglobulin—like receptors, killer cell immunoglobulin-like receptor 2D long 1(KIR2DL1), killer cell immunoglobulin-like receptor 3D long 1, and natural killer group 2 member A(NKG2A) [10, 12–17], and decreased expression of activating receptors, including natural killer group 2D(NKG2D), natural cytotoxicity receptor 1(NKP46), and natural killer cell protein 30 [11, 18, 19]. The survival of EECs is made possible by the reduced NK cell toxicity shown by the increased expression of inhibitory receptors and lower expression of activating receptors on NK cells from EMS patients. Additionally, there was less NK cell chemotaxis in the peritoneal cavity of EMS patients. Following menstruation, NK cell chemotaxis rises in healthy women. However, during the menstrual cycle, NK cell chemotaxis in EMS patients stays low [20]. The reduced expression of the particular chemokine (c-x3-c motif) ligand 1 on NK cells in the peritoneal cavity of EMS patients may be the cause of this [21]. Together with NK cells, macrophages are immune troops that eliminate aberrant cells early on. The immunological escape of EECs is also made possible by the compromised phagocytic ability of macrophages in EMS patients. In individuals with EMS, for instance, the peritoneal cavity has reduced expression of CD36, a marker of phagocytosis [22]. This alteration is associated with increased autophagy in macrophages and a reduction in hematopoietic cell kinase [23].

NK cells kill cells in a different way than CD8 + T cells do. Antigen-presenting cells must release particular antigen peptides for CD8 + T cells to carry out targeted immunological damage. There is an increase in CD8 + T cell concentrations in the peripheral blood of healthy women during the luteal phase. However, EMS patients did not exhibit this alteration [24]. EMS patients’ peripheral blood also had fewer natural killer T(NKT) cells [25]. Moreover, toxicity was decreased, CD8 + T cells’ capacity to generate perforin was diminished in EMS patients’ menstrual blood [26]. All of these imply that the immunological escape of EECs may be caused by a reduced ability for clearance and a reduction in the quantity of CD8 + T cells. Additionally, dendritic(DC) cells are necessary for CD8 + T cells to deliver antigens. Throughout the menstrual cycle, EMS patients’ endometrium showed markedly lower levels of CD83 expression, a hallmark of plasmacytoid DC cells development [27]. Lysosomal-associated membrane protein (LAMP) can assist DC cells in presenting and absorbing the antigen. Women with EMS had fewer LAMP + DC cells in their endometrium during the menstrual and proliferative phases. In contrast, women without EMS had more LAMP + DC cells in their endometrium during the secretory and menstrual phases [28]. This suggests that EMS patients have a lower number of mature DC cells in their menstrual and proliferative endometrium, and that these cells’ capacity to deliver antigen is compromised, which impacts CD8 + T cells’ ability to eliminate EECs. Because of the invisible character of EMS’s early onset, it is impossible to say with certainty if immune killer cell failure predates EMS’s onset.

## EECs impair the function of immune killer cells

EECs’ capacity to prevent immune killer cells from doing their job is the reason they can evade immune killing. EECs can secrete a range of cytokines to prevent NK cells from doing their job. For instance, EMS patients have higher levels of interleukin(IL)-6 in their peritoneal cavity, and IL-6 can inhibit NK cells’ Src homology phosphotyrosyl phosphatase 2 (SHP-2). When SHP-2 is down-regulated, NK cell toxicity is decreased because SHP-2 is a signaling switch that allows NK cells to accept instructions and carry out attacks [9]. Compared to eutopic stromal cells, endometriotic stromal cells generate higher IL-6 [29]. Furthermore, EECs can produce interleukin-15 (IL-15), which can encourage endometriotic cell proliferation and invasion. While IL-15 can encourage NK cell growth and differentiation, it can also prevent NK cells from producing granzyme and interferon-γ(IFN-γ) and from expressing the activation receptors NKG2D and natural cytotoxicity receptor 44, thereby reducing NK cell cytotoxicity [30]. It was in line with the observation that, following the initiation of EMS, NK cells rose but NK cytotoxicity fell. EECs also sercrete the immunosuppressive substance transforming growth factor -β(TGF-β) [31, 32], which directly or indirectly decreases the expression of the activating receptor NKG2D on NK cells [33]. Additionally, TGF-β signaling suppresses important metabolic processes that are controlled by mammalian target of rapamycin(mTOR), which in turn suppresses the growth and cytotoxicity of NK cells [34]. EECs-derived exosomes can inhibit NK cell toxicity in addition to cytokines. EECs-derived exosomes include a range of immunosuppressive substances that can inhibit NK cells’ activation receptor NKG2D and lessen their cytotoxicity [35].

Furthermore, EECs inhibit macrophage phagocytosis, while macrophages support EEC survival. EECs increase the synthesis of prostaglandin E2 (PGE2). PGE2 can prevent peritoneal macrophage phagocytosis by suppressing macrophage expression of matrix metalloproteinase(MMP) 9, CD36, and annexin A2, which are linked to PGE2’s activation of the macrophages’ prostaglandin E receptor 2 signaling pathway [36–39]. A rise was observed in the expression of hypoxia inducible factor-1 alpha (HIF-1α). HIF-1α can increase the expression of miRNA-20α, decrease the expression of dual specificity phosphatase 2, and encourage the activation of extracellular regulated protein kinases. This causes EECs to express cyclooxygenase-2 (COX-2) [40, 41]. Given that COX-2 is the precursor of PGE2, hypoxia may have an indirect impact on macrophage phagocytosis of EECs. EECs are only impacted by estrogen when they are in the proliferative phase. EECs’ CD200 expression and peritoneal macrophages’ CD200R expression were both elevated by estrogen [42]. However, CD200R may prevent macrophage phagocytosis. Thus, in the estrogen environment, EECs can employ CD200R to prevent macrophage phagocytosis. In contrast to normal EECs, Mei et al. [43]. discovered that EECs prevented macrophage phagocytosis. The EECs’ production of indoleamine 2,3-dioxygenase 1 (IDO1) may have contributed to the reduction in macrophages’ phagocytic capacity [44]. It’s interesting to note that suppressing macrophage activity might encourage EECs’ growth. Macrophages were able to generate more IL-6 when they were co-cultured with EECs. In EECs, IL-6 triggers the c - jun n - terminal kinase pathway to produce chemokine (c-c motif) ligand 17(CCL17), whereas in macrophages, the CCL17-C-C chemokine receptor type 4(CCR4) axis triggers the nuclear factor kappa B(NF-κB) pathway to produce IL-6 [45]. Because of this vicious cycle, there may be an excess of IL-6 in the peritoneal microenvironment, which might encourage the adhesion, angiogenesis, and survival of EECs [46–48].

EECs can inbibit CD8 + T cell death and dendritic cell maturation. EECs that overexpress HSD11B1 may produce more cortisol, thereby inhibiting dendritic cells from maturing. Similarly, myeloid dendritic cell maturation was decreased and HSD11B1 was increased in endometriotic lesions [49]. Antigen presentation and CD8 + T cell killing against EECs are both impacted by decreased dendritic cell maturation. EECs can also cause CD8 + T cell dysfunction by upregulating the signal transducer and activator of transcription 1(STAT1)/programmed cell death protein 1(PD-1) pathway and metabolic reprogramming facilitated by glycolysis [50]. EECs-derived exosomes can also prevent T lymphocytes from dying by blocking the fas ligand(FASL) and tumor necrosis factor - related apoptosis - inducing ligand pathways [35]. In conclusion, there is a strong association between the suppression of EECs and the dysfunction of immune killer cells.

## Menstrual components can aid EECs in escaping immune

**Fig. 1:**
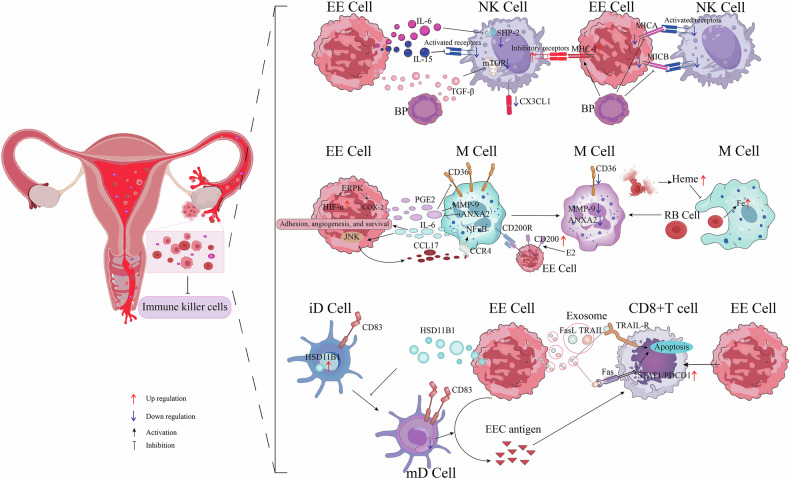
The cellular and molecular mechanisms of immune escape in ECC. The figure describes the cellular and molecular mechanisms of immune escape in EECs, mainly including: 1. EECs inhibit NK cells through secreting inhibitory factors and direct contact; 2.EECs inhibit macrophages through the secretion of inhibitory factors and direct contact, as well as by suppressing them with menstrual components. 3. EECs inhibit DC cells maturation and simultaneously suppresses CD8 + T cells.

Other components of menstrual reflux into the abdominal cavity will also prevent the immunological killer cells in the abdominal cavity from doing their job. Platelets in menstrual component, for instance, can reduce NK cells toxicity. During heavy bleeding, platelets might stop the endometrial shedding. Nevertheless, platelet-produced TGF-β1 can decrease NK cells’ activating receptor NKG2D and prevent NK cell proliferation, cytotoxicity, and immune factor release [51–54]. Further, EECs release thrombin and thromboxane A2, which activate platelets [55]. When NK cells and platelets were co-cultured, the toxicity of NK cells was also considerably decreased. Instead of NK cell apoptosis, this decrease in lethal toxicity was brought about by inhibition of NK cell degranulation, decreased expression of the activating receptors NKG2D and NKP46 in NK cells, and decreased production of IFN-γ [56]. Furthermore, major histocompatibility complex class I(MHC-I) expression in EECs can be enhanced by platelets, but major histocompatibility complex class I chain-related protein A(MICA) and major histocompatibility complex class I chain-related protein B(MICB) expression can be inhibited. MICA/B can attach to NKG2D to increase NK cell toxicity, while MHC-I can bind to NK cells’ inhibitory receptors to reduce NK cell toxicity [56]. This implies that EECs may be able to evade NK killing with the aid of platelets. Heme is released during the clearance the red blood cells. Patients with EMS also have a greater heme content in their abdominal cavity. Heme levels that are too high prevent macrophages from being phagocytic [57]. When menstruation reflux occurs, the phagocytization of red blood cells by macrophages results in iron overload, which damages the macrophages and induces oxidative stress. Eventually, this reduces the ability of the macrophages to phagocytize EECs [58–60]. During menstruation, macrophages can release MMPs, including MMP12, MMP9, and MMP14, which are essential for the endometrium’s disintegration [61]. These MMPs might make it possible to plant EECs in the peritoneal space. MMP27 can break down the extracellular matrix and aid in the invasion of aberrant cells, and its expression is highest in the endometrium during menstruation [62]. It is still necessary to confirm if MMP27 plays a role in the implantation of EECs in the ovary and peritoneum. Figure 1 depicts the molecular and cellular mechanisms by which EECs escape the immune system.

## Pro-inflammatory immune microenvironment in EMS shifts to an anti-inflammatory state

### The immunological microenvironment in EMS is pro-inflammatory in the early stages but shifts to an anti-inflammatory in the later stages

As aberrant cells, EECs proliferate in the peritoneal cavity and then cause an immunological reaction. However as the lesion continues to spread, the immune microenvironment progressively shifts from a pro-inflammatory to an anti-inflammatory state. In addition to their potent phagocytic ability, M1 macrophages can cause inflammation by releasing proinflammatory substances such as tumor necrosis factor—alpha(TNF-α), IL-1β, IL-6, and IL-12. Additionally, cxc and cc chemokines are generated to draw in T cells and neutrophils, which progressively escalate inflammation [63, 64]. M2 macrophages can release inhibitory inflammatory molecules, including TGF-β and IL-10, but they have a limited phagocytic ability. Additionally, they releases CCL17 and c-c motif chemokine 22(CCL22) to decrease excessive inflammation and attract immunosuppressive cells [65]. According to Lagana et al. [66], M1-type macrophages gradually decreased in EMS lesions from stage I to stage IV, while M2-type macrophages exhibited the reverse tendency. The immune microenvironment in the peritoneal cavity would shift from pro-inflammatory to anti-inflammatory as EMS progressed, according to Nie et al., further demonstrated who further demonstrated that the ratio of M2/M1 macrophages in the peritoneal fluid of patients with III-IV was higher than that of patients with I-II. Nie et al. also discovered that patients with III-IV had a higher ratio of M2/M1 macrophages in their peritoneal fluid compared to patients with I-II. This further supported the idea that as EMS progresses, the immune microenvironment in the peritoneal cavity will shift from pro-inflammatory to anti-inflammatory [67]. Animal models showed similar patterns. On days four and seven following endometrial tissue transplantation into mice, M1 macrophages’ expression of inducible nitric oxide synthase(iNOS) peaked. On days 10 and 14, the expression of iNOS progressively declined. The highest level of CD204 expression, a marker of M2 macrophages, was observed on day 14 following transplantation [68]. Yuan et al. demonstrated that as the lesions developed, the number of macrophages in the peritoneal cavity of mice with EMS dropped and displayed M1-type function. In contrast to the M1 type, macrophages moving from bone marrow to the peritoneal cavity displayed the M2 type. This implies that the peritoneal immunological microenvironment may be altered by immune cells from other places [69]. However, more research is necessary to determine the exact mechanisms by which immune cells from other sites produce pro- or anti-inflammatory effects. According to a meta-analysis, M1 macrophages predominated in the eutopic endometrium of EMS patients in stages I–II, whereas M2 macrophages predominated in the eutopic endometrium of EMS patients in stages III–IV [70]. The immunological microenvironment of EMS is transitioning from early proinflammatory M1-type macrophages to late M2-type macrophages, as evidenced by these characteristics.

In the immunological response, macrophages and T helper cells typically work together. M1 macrophages, for instance, can release factors to Promote T helper 1(Th1) cell polarization, while M2 macrophages can release TGF-β and IL-10 to promote Th2 cell polarization [71, 72]. IFN-γ produced by Th1 cells can then control typical M1 polarization, whereas IL-4 and IL-13 produced by T helper 2(Th2) cells can also cause M2 activation of macrophages [73]. Th1 cells predominate in the peritoneal fluid during the early stages of EMS, while Th2 cells do so in the latter stages [74]. Patients with advanced EMS had particularly elevated levels of IL-4, a cytokine of the Th2 immune response, in their peritoneal fluid, which suggests that anti-inflammatory Th2 cells predominate in the latter stages of EMS [75, 76]. Similarly, regulatory T(Treg) cells with immunosuppressive function were considerably higher in the peritoneal fluid of patients with advanced EMS. EMS increases the number of Treg cells in the peritoneal cavity, particularly the fraction of CD25+forkhead box P3 protein+ Treg cells, which a potent immunosuppressive effect [77, 78]. The induction of Treg cell maturation is dependent primarily on TGF-β. In women with early-stage EMS, immunological proinflammatory responses predominate, and low levels of TGF-β in the peritoneal fluid may be the cause of fewer Treg cells [79]. Li et al. [80] further showed that as EMS phases advanced, peritoneal fluid’s Treg cells and TGF-β levels steadily rose. In general, the proportion of Treg cells to T helper 17(Th17) cells is balanced. Th17 cells can stimulate inflammation, while Treg cells can suppress it. The function of Th17 cells may shift even though the current study demonstrates that as the peritoneal cavity’s Th17 cell proportion steadily rises as the EMS lesion worsens [81]. Although Th17 cells secrete IL-17A, patients with advanced EMS had lower levels of IL-17A in their peritoneal fluid than those in the early stages [78]. In addition to being pro-inflammatory, Th17 cells also generate IL-10. For instance, through v - maf avian musculoaponeurotic fibrosarcoma oncogene homolog/retinoic acid receptor-related orphan receptor/B lymphocyte-induced maturation protein 1 signaling, IL-27 generated by macrophages and EECs can cause Th17 cells to create IL-10, hence having an anti-inflammatory impact [82]. This also explains why, despite a proportional increase in Th17 cells, the immune microenvironment in advanced EMS is still dominated by anti-inflammation. This is because Th17 cells generate IL-10, which has anti-inflammatory qualities, and less IL-17A. According to the data above, EMS patients’ immune microenvironment shifts from an early, pro-inflammatory state to a late, anti-inflammatory state. It is important to note that although the immune microenvironment in the late stage primarily works to reduce inflammation, this does not imply that the lesion’s inflammation is less severe than it was in the early stages.

## Instead of removing the lesion, the proinflammatory microenvironment accelerates the development of EMS

The proinflammatory state of the immune microenvironment is a double-edged sword. While pro-inflammatory factors may enhance the killing of EECs, they can also promote the progression of EMS. Although IL-2 can activate NK cells to increase their toxicity and support Th1 cell survival and differentiation, it can also stimulate EECs’ proliferation and invasion [83, 84]. Nevertheless, it remains unclear how much IL-2 is present in the abdominal cavity in the early and late phases [85]. Th17 cells can be activated and inflammation can be encouraged by IL-1β [86]. However, IL-1β can also upregulate vascular endothelial growth factor(VEGF) and COX-2 expression, promote EEC migration, and enhance the growth and further implantation of endometriotic lesions [87, 88]. Activated Th17 cells can also release IL-17A, which can boost EECs’ production of the inhibitory ligand HLA-G and shield them from NK killing [89]. Even though IL-8 can cause neutrophils to differentiate, it can also increase the expression of B-cell lymphoma 2 and survivin in EECs via the phosphatase and tensin homolog deleted on chromosome ten/protein kinase B(AKT) pathway, prevent apoptosis, and promote cell proliferation [90, 91]. When M1-type macrophages are cultivated alongside endometrial stromal cells, they can activate the macrophages’ inflammasome, which increases IL-1β release and may worsen endometriotic cell migration [87]. When NK cells and EECs are co-cultured, the NK cells cause the EECs to produce more IL-22 and chemokine (c-c motif) Ligand 2(CCL2), which attracts more macrophages. EECs can produce more CCL2 when exposed to IL-22. Despite being a pro-inflammatory agent, CCL2 can stimulate EECs invasion and proliferation via the mitogen-activated protein kinase/extracellular signal-regulated kinase1/2 and AKT pathways [92]. Furthermore, estrogen acts on mast cells by activating inflammasomes, which increases the release of IL-1β [93]. Mast cells can boost the immune response, but the inflammation they cause can also accelerate the course of EMS. It is important to keep in mind that EMS inflammation will only get worse. For instance, as EMS advances, the amounts of IL-8 and IL-1β in EMS patients’ abdominal cavities will keep rising [87]. The proinflammatory microenvironment, however, was never able to eradicate EECs. The body activates immunosuppressive cells to regulate inflammation, thereby limiting its continuous increase and preventing chronic inflammation. IFN-γ and IL-6, two immune proinflammatory cytokines, activate Treg cells to generate fibrinogen-like protein 2(FGL2) via the STAT1 pathway. To establish chronic inflammation, the pro-inflammatory immunological microenvironment of EMS must transition to an anti-inflammatory state, which is shown by the fact that FGL2 can stimulate M2 macrophage polarization and Th2 differentiation [94].

## Recurrent bleeding and lesion progression induce an anti-inflammatory change in the immune microenvironment

EECs can be attacked by immune killer cells, but they cannot be completely eliminated; therefore, EMS will continue to progress. By attracting immunosuppressive cells, EMS reduces the death of immune killer cells as the disease progresses. C-x-c Motif Chemokine Ligand 8(CXCL8), which is consistently expressed at high levels in EECs, attracts macrophages and promotes their M2 polarization [95, 96]. Similarly, macrophages can be recruited and polarized into M2 type by colony-stimulating factor 1 and CCL2, which are produced by nerve fibers in the lesion [97, 98]. MCP1 and CCL5 produced by endometriotic cells also recruit macrophages [99–101]. EECs’ production of lactic acid and IL-6, as well as exosomes’ miR-301a-3p and miR-146a-5p, can lead macrophages to M2 polarize [102–104]. Moreover, the cytokines secreted by immunological proinflammatory cells can polarize macrophages into the M2 type when EMS lesions progress. For instance, Th17-produced IL-17A can accelerate the growth of lesions by boosting both angiogenesis and macrophage M2 polarization. Additionally, EMS patients’ plasma and lesions showed a substantial rise in IL-17A [105]. These findings imply that more macrophages are drawn to the EMS lesion as it develops, but that these macrophages are oriented toward the anti-inflammatory M2 type. The inflammation brought on by the development of EMS also attracts Treg cells. For instance, EECs’ CCL22 can attract Treg cells; this may be because CCL22 binds to the CCR4 receptor on Treg cells [106, 107]. Treg cells may be recruited via the CCL20-CCR6 pathway, as CCL20 levels are elevated in the peritoneal fluid of EMS patients. This would increase the proportion of Treg cells in EMS patients’ peritoneal fluid [108, 109]. Treg cell migration from the peripheral blood may explain why advanced EMS patients exhibit a higher Treg cell proportion in peritoneal fluid but a lower proportion in peripheral blood compared to non-EMS women [109]. Additionally, EMS lesions show greater Treg cell infiltration than eutopic endometrium [110]. While immunosuppressive cells can reduce inflammation, their recruitment to EMS lesions may impair immune killer cell-mediated EEC destruction, thereby promoting EMS progression. While immunosuppressive cells can reduce inflammation, their recruitment to EMS lesions may impair immune killer cell-mediated EEC destruction, thereby promoting EMS progression.

Despite not being in the uterus, the EMS lesions can react to the cyclical changes in progesterone and estrogen, resulting in the EMS lesions bleeding regularly. When EMS lesions bleed periodically, EECs may release alarm proteins such as thymic stromal lymphopoietin(TSLP), IL-33, and IL-25 [111]. These alarmins can induce an anti-inflammatory shift in the immune microenvironment. Patients with EMS exhibited significantly higher IL-25 levels in peritoneal fluid than healthy controls, independent of disease severity [112]. EMS patients’ peritoneal fluid, plasma, and lesions all have high levels of IL-33, which causes pain [113, 114]. TSLP expression is significantly upregulated in EMS lesions, and pro-inflammatory IL-1β can further stimulate TSLP secretion by EECs [115, 116]. All of these show that EMS hemorrhage regularly causes alarmin levels in the EMS lesion microenvironment to rise. This, in turn, causes the immune microenvironment to shift in an anti-inflammatory manner. For instance, TSLP, IL-25, and IL-33 all stimulate the growth and differentiation of Treg cells, polarize macrophages to the M2 type, and increase the quantity of Th2 cells [117–124]. Proinflammatory immune cells are also suppressed by Treg cells, Th2 cells, and M2-type macrophages. For instance, Th2 cell-derived IL-4 and IL-13 can prevent Th1 cells from acting as immune cells [125]. Treg cells can generate FGL-2, stimulate the generation of Th2 cytokines, and suppress the Th1 and Th17 cell-mediated immune response [126]. Repeated lesion bleeding can activate platelets,which then stimulate EECs to produce more estrogen,thereby inducing TSLP secretion [127, 128]. Furthermore, platelet-activating factor can also be secreted by EECs [55]. The TSLP receptor is also found in platelets, and TSLP can activate platelets, which leads to recycling [129]. Studies suggest that reducing platelet levels may halt EMS progression [130]. This implies that preventing the interaction between alarmin and platelets can stop EMS from progressing. Platelets can also release anti-inflammatory cytokines thereby suppressing immune responses. Platelets can generate TGF-β, suppress Th1 cells and M1 macrophages, stimulate Treg cells and M2 macrophages, and change the immune system into an anti-inflammatory state [131–135].

## An anti-inflammatory microenvironment exacerbates fibrotic and angiogenic processes in EMS lesions

**Fig. 2:**
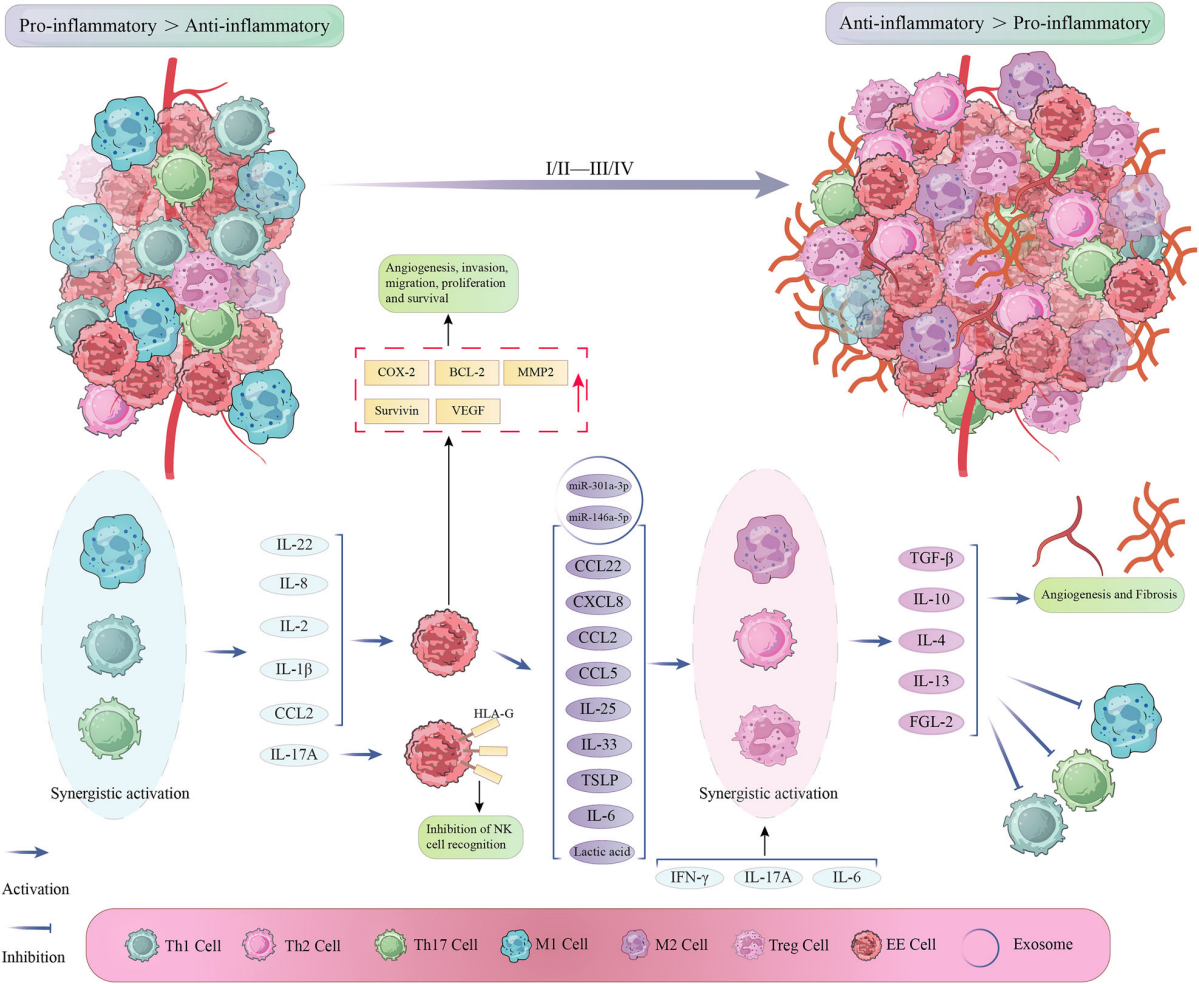
Changes from the EMS immune pro-inflammatory microenvironment to the immune anti-inflammatory microenvironment. The figure describes the changes in the immune microenvironment of EMS lesions from early lesions to late lesions. The lesions show that immune pro-inflammatory cells are dominant in early lesions, while immune anti-inflammatory cells are dominant in late lesions. The pro-inflammatory factors released by immune cells such as Th1, Th17, and M1 promote the growth, angiogenesis, migration, and invasion of EECs, leading to its continuous growth. EECs releases recruitment factors and alarm proteins to promote the activation of immune anti-inflammatory cells, thereby inhibiting immune pro-inflammatory cells. Immunopro-inflammatory cells can also promote angiogenesis and fibrosis in the lesion.

Allthough the immunosuppressive microenvironment prevents excessive inflammatory in the lesion, it paradoxically creates conditions for EMS progression. Angiogenesis in EMS lesions can be stimulated by immunological anti-inflammatory cells, on the one hand. For instance, M2 macrophages infiltrating the lesions can encourage angiogenesis [136]. This pro-angiogenic effect could be linked to M2 macrophages’ IL-10 and IL-4 production. Through the IL-10R/STAT3 pathway, IL-10 can cause vascular endothelial cells to express more VEGFA and MMP2 [137, 138]. Likewise, the production of IL-10 by immature DC cells in EMS lesions stimulates angiogenesis [138]. Immature DC cells in EMS patients’ peritoneal fluid may steadily rise as the illness worsens, according to research by Tariverdian et al. This could imply that when the illness grows more serious, immature DC cells also support immunological anti-inflammation [139]. The fibrosis of EMS lesions is also encouraged by the immunological anti-inflammatory microenvironment. Th2 cells, Treg cells, and M2 macrophages can all produce immunosuppressive substances including IL-10, IL-4, and TGF-β [140]. Among these, TGF-β is the primary agent that promotes the fibrosis of EMS lesions, and EMS patients’ abdominal cavities also have elevated levels of TGF-β [141]. Through the TGF-β signaling pathway, TGF-β can induce endometrial stromal fibroblasts to produce collagen, which in turn promotes fibrosis [142]. Additionally, the peritoneal fluid of EMS patients contains higher levels of Sphingosine 1-phosphate, which can encourage macrophage M2 polarization and boost IL-6 production. In EECs, IL-6 can also result in the ongoing activation of the STAT3 and NF-κB pathways, which can lead to fibrosis and collagen formation [143, 144]. EMS patients’ abdominal hypoxia can potentially change the immune microenvironment to be more anti-inflammatory. Through HIF-α, hypoxia can cause EECs to release semaphorin 3A(SEMA3A). SEMA3A then encourages M2 polarization of macrophages, which furthers the fibrosis of EMS lesions [145]. Figure 2 illustrates the process by which the EMS immune microenvironment changes from an early pro-inflammatory to a late anti-inflammatory state.

## During the latter stages of EMS, immune killer cell exhaustion occurs

### Immune killer cell exhaustion is present in the EMS microenvironment

During EMS, immune killer cells do not always carry out their normal killing activity. Immunosuppressive cells and EECs will continuously suppress immune killer cells as the disease worsens, eventually wearing them out. According to a single-cell sequencing study, EMS patients’ ectopic tissues have markedly elevated NK and T cell exhaustion ratings, as well as an increase in the inflammation score [146, 147]. It serves as a reminder that despite the presence of inflammation in EMS lesions, immune killer cells are worn exhausted and unable to eliminate EECs [147]. Particularly in patients at stage IV, the percentage of PD-1 + NK cells in the advanced peritoneal fluid of EMS patients is markedly elevated [148]. One indicator of NK cell exhaustion is PD-1. NK cells’ activity is hindered when the PD-1/programmed cell death - ligand 1(PD-L1) pathway is activated, which reduces their capacity to kill [149]. The percentage of NK cells in EMS patients’ peritoneal fluid that express the inhibitory receptor killer cell immunoglobulin-like receptor 2D long 1(KIR2DL1) continues to rise as disease stages worsen, while NK cell toxicity continues to decline [16, 150]. The depletion trend of T cells with cytotoxic potential was comparable to that of NK cells. In EMS patients’ eutopic and ectopic endometrial tissues, the phenomenon of CD8 + T cell exhaustion was markedly elevated [147]. The CD8 + T cells in the abdominal cavity of EMS patients express more CD38 than those in women without EMS [151]. CD38 is a sign of CD8 + T cell exhaustion, and elevated CD38 expression frequently denotes worsened DNA damage in CD8 + T cells [152]. In the peripheral blood and abdominal cavity of EMS patients, the expression of the exhaustion factor PD-1 on CD8 + T cells is also trending upward, which causes the T cell signaling pathway on CD8 + T cells to activate continuously, ultimately leading to exhaustion [153, 154]. As the illness stage advances, the loss of CD8 + T cells will also worsen. The peripheral blood’s CD8 + CD56 + T cells, which have both NK cell and CD8 + T cell traits, will progressively decline as the disease grade increases [8]. Furthermore, NKT cells exhibit a marked exhaustion in EMS lesions, particularly in deeply infiltrating EMS. T cell exhaustion can be induced by both Lymphocyte Activation Gene 3 and Cytotoxic T-Lymphocyte Antigen 4, and NKT cells express these proteins at higher levels [155–157]. NKT cell counts declined in EMS patients’ peripheral blood and peritoneal fluid, and this decline was inversely connected with disease stage. In the meantime, immune killer cells’ IFN-γ exhibited the same pattern as NKT [158]. IFN-γ can stimulate NK cells, increase their capacity to kill, and simultaneously encourage CD8 + T cell proliferation and differentiation, guaranteeing CD8 + T cells’ regular immune response [159, 160]. Like IFN-γ, TNF-α is generated by immune killer cells and can activate CD8 + T cells and NK cells [161, 162]. Similar to IFN-γ, TNF-α levels in the abdominal cavity are high in patients’ early stages and decrease in their later stages [163]. To a certain extent, the capacity of immune killer cells to kill is represented by IFN-γ and TNF-α. Early EMS patients have higher quantities of these substances in their peritoneal fluid, while late EMS patients have lower levels. This also suggests that the immune system’s capacity to eliminate the lesion is gradually depleted as it spreads.

## The exhaustion of immune killer cells in EMS may result from the immunological anti-inflammatory microenvironment

**Fig. 3:**
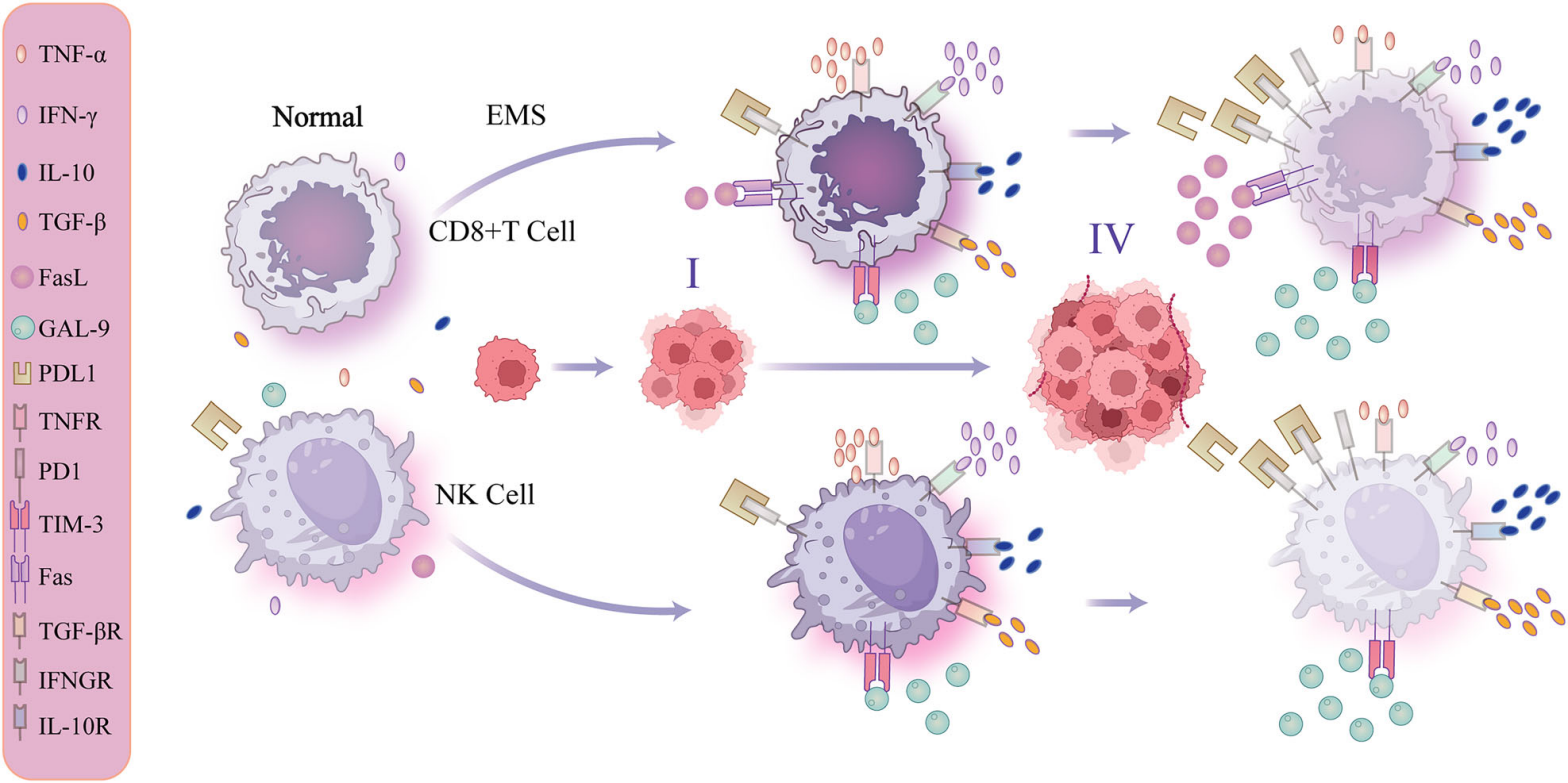
shows that as the disease progresses, cytokines that cause the depletion of NK cells and CD8 + T cells, such as IL-10, TGF-β, and PDL1, keep increasing, while IFN-γ and TNF-α, which activate immune killer cytokines, keep decreasing.

Immunosuppressive cells generate TGF-β and IL-10, which both can suppress immunological killer cells and cause exhaustion. The largest concentration of IL-10 in peritoneal fluid occurs in the late stages of EMS [83]. The findings of studies on NK cell exhaustion brought on by IL-10 are largely consistent [164]. NK cell exhaustion results from IL-10’s upregulation of NKG2A on NK cells [165]. Nevertheless, recent studies suggest that IL-10 has a dual effect on CD8 + T cell exhaustion. IL-10 can cause CD8 + T cell exhaustion in organ transplant recipients [166]. Blocking IL-10 during intestinal ischemia-reperfusion can boost the release of immune killer factors and restore CD8 + T cell exhaustion [167]. IL-10 may help restore the metabolic reprogramming of exhausted CD8 + T cells in the context of oncology studies [168]. TGF-β has long been thought of as a negative regulatory factor for NK cells since it can activate P35 and EEF2K in NK cells, decrease oxidative phosphorylation, lower mitochondrial quality, and reduce mTOR expression, all of which can result in NK cell exhaustion [169–171]. The exhaustion of CD8 + T cells is likewise significantly influenced by the same TGF-β. On the one hand, TGF-β can cause SMAD2/SMAD3 to become activated, which will deplete CD8 + T cells. It can also prevent stem cell-like CD8 + T cells from proliferating and prevent CD8 + T cells from being replaced [172–175]. On the other hand, TGF-β can impact metabolism by blocking the mTOR pathway in CD8 + T cells [176]. EMS patients’ peritoneal fluid has a markedly high level of TGF-β, which progressively rises as the condition worsens [163]. This suggests that the immune killer cell depletion brought on by immunological anti-inflammatory substances is more severe in the advanced stage of EMS. Studies conducted in vitro have demonstrated that when NK cells, macrophages, and EECs were co-cultured, the co-culture system’s production of cytotoxic factors, perforin and IFN-γ, was considerably decreased, whereas IL-10 and TGF-β increased [177]. This implies that the immune system’s anti-inflammatory microenvironment may cause NK cells to become incapable of eliminating EECs. One important molecule that suppresses CD8 + T cells is PD-L1, which also activates surface PD-1 and limits the mitochondrial energy metabolism of CD8 + T cells, ultimately resulting in exhaustion [178]. The production of PD-L1 can stimulate by immunosuppressive cells in an immunological anti-inflammatory microenvironment. M2 macrophages, for example, can increase PDL1 expression [40]. This is further supported by the fact that the degree of PD-L1 in the abdominal cavity of EMS patients is positively connected with the severity of the illness [179]. Furthermore, a variety of cytokines have the ability to induce immune killer cell death. FASL, which is produced by EECs in response to pro-inflammatory stimuli, can attach to T lymphocytes’ FAS receptor and trigger apoptosis [180]. Patients with advanced EMS also had considerably higher levels of soluble FASL in their peritoneal fluid when compared to those with early EMS [181]. Galectin - 9(GAL-9) can inhibit Th1 cell responsiveness by binding to the TIM-3 receptor. Low concentrations of GAL-9 can cause activated T cells to release cytokines, whereas high concentrations can cause CD8 + T cells to undergo apoptosis [182]. With the progression of EMS disease, peripheral blood levels of GAL-9 rise, which could be connected to the shift from an immune-pro-inflammatory to an immune-anti-inflammatory microenvironment [183]. Although NK cells also express the TIM-3 receptor on their surface [184], more evidence is still needed to confirm that GAL-9 causes CD8 + T and NK cell exhaustion in EMS. Figure 3 illustrates the precise process through which immune killer cell depletion worsens as EMS advances.

## The immune miroenvironment provides insights into effectively treating EMS

One way to treat EMS is to increase the toxicity of immune killer cells. However, immune killer cells are inhibited by the EMS immunological anti-inflammatory microenvironment. Therefore, one approach to treating EMS may be to remove this restrictive environment without producing severe inflammation. Melioli et al. [185]. discovered that recombinant IL-2 might partially repair the cytotoxic damage to CD8 + T cells in EMS patients. When recombinant IL-2 was administered to EMS rats, the lesions considerably decreased, and the quantity of macrophages, NK cells, and dendritic cells in the implants rose [186]. Likewise, in EMS mice, IL-12 can increase the toxicity of NK cells, which lowers the size of EMS lesions [187]. These findings suggest that one potential treatment option for EMS is to increase the toxicity of immune killer cells. DC cells are antigen-presenting cells; however, iDC cells make up a larger percentage of EMS lesions and are typically involved in immunosuppression and angiogenesis promotion [138, 188]. The percentage of mature DC cells in the abdominal cavity rose after EMS mice were given lipopolysaccharide with pro-inflammatory effects, although the size and mass of EMS lesions shrank [189]. By preventing STAT3 phosphorylation, encouraging DC cells maturation, and raising the Th1/Th2 cell ratio, RHIL-37 can stop the lesion from getting worse [190]. One potential avenue for EMS treatment is to encourage dendritic cell maturation and change the direction of the immune environment. Pro-inflammatory responses may aid DC cells maturation. Inflammation is also present in EMS lesions, but it prevents DC cells from maturing. We still need to confirm whether this is due to a larger level of immunological anti-inflammation than immune pro-inflammation. To some extent, the immunological microenvironment characterized by immunosuppression can be relieved by removing the contact between alarm proteins and platelets. EMS fibrosis and the epithelial-mesenchymal transition can be stopped by removing platelets from the lesion because this will decrease the production of the alarm protein TSLP, which will decrease the aggregation of M2-type macrophages, Th2 cells, and Treg cells while increasing the aggregation of Th1 cells [130].

Moreover, the immunological abnormalities of EMS do not appear to have been altered by the surgery. Using EMS patients’ peripheral blood as an example, the percentage of NK cells expressing KIR2DL1 did not differ between EMS patients prior to and during the procedure [13, 191]. Following EMS surgery, KIR2DL1, an inhibitory receptor for NK cells, has not improved, suggesting that the procedure may not restore immune function to normal levels. Immune cell activity and quantity in the abdominal cavity and peripheral blood increase following gonadotropin-releasing hormone intervention, and postoperative immune cell activity is correlated with the recurrence rate, which offers insights into the mechanism of recurrence following EMS [192–194].

## Summary

Our analysis indicates that the immune response of EMS is classified as follows: lesion formation following immune escape, dominance of pro-inflammation immune microenvironment, dominance of anti-inflammation immune microenvironment, and exhaustion of immune killing in the late microenvironment. Researchers remain uncertain whether immunological abnormalities lead to EMS or if EMS itself causes immune abnormalities due to the complex nature of EMS development. We find it challenging to investigate the immuno-inflammatory microenvironment of early EMS lesions due to the lack of EMS diagnostic techniques and the decision to use early medication treatment. It is important to remember that while the advanced lesion of EMS mainly consists of an anti-inflammatory and immunological microenvironment, pro-inflammatory elements are still present. Furthermore, the EMS immunological microenvironment should alter between the proliferative and secretory stages since estrogen and progesterone affect women’s immune cells. The majority of research, however, has not addressed the influence of the menstrual cycle. We still need to learn more about the intricate relationships between numerous immune cells in the complicated immunological microenvironment of EMS.
